# Examining the impact of a Mindfulness-Based Stress Reduction intervention on the health of urban South Africans

**DOI:** 10.4102/phcfm.v10i1.1614

**Published:** 2018-06-18

**Authors:** Simon L. Whitesman, Michelle Hoogenhout, Linda Kantor, Katherine J. Leinberger, Anik Gevers

**Affiliations:** 1 Institute for Mindfulness South Africa, Cape Town, South Africa; 2 Division of Family Medicine and Primary Care, Faculty of Medicine and Health Sciences, University of Stellenbosch, South Africa; 3 Department of Paediatrics and Child Health, Red Cross War Memorial Children’s Hospital, University of Cape Town, South Africa; 4 Graduate School of Business, University of Cape Town, South Africa; 5 Adolescent Health Research Unit, Department of Psychiatry, University of Cape Town, South Africa

## Abstract

**Background:**

Mindfulness-based stress reduction (MBSR) has been found to have significant health benefits in studies conducted in the global North.

**Aim:**

This study examined the effects of MBSR on stress, mood states and medical symptoms among urban South Africans to inform future research and clinical directions of MBSR in local settings.

**Setting:**

Participants completed an 8-week MBSR programme based in central Cape Town.

**Method:**

A retrospective analysis of 276 clinical records was conducted. Mindfulness, stress, negative and positive mood, medical symptoms and psychological symptoms were assessed before and after the intervention using self-report questionnaires. We compared pre and post-intervention scores and examined the relationship between changes in mindfulness and changes in stress, mood and medical symptoms.

**Results:**

Mindfulness scores were significantly higher after intervention, both on the Kentucky Inventory of Mindfulness Skills (KIMS) and the Mindful Attention Awareness Scale (MAAS). Changes on the KIMS were associated with reductions in stress, negative mood, psychological symptoms and total medical symptoms, and improvement in positive mood. Changes in mindfulness, as measured by the MAAS, were significantly correlated only with reduced total number of medical symptoms.

**Conclusion:**

This study provides preliminary evidence for the positive health impact of MBSR on urban South Africans, and in turn acceptability and feasibility evidence for MBSR in South Africa and supports the case for larger trials in different local settings.

## Introduction

Mindfulness is the awareness that arises when paying attention intentionally, without judgement, to the phenomena (thoughts, feelings and sensations) moving through the present moment in service of insight, wisdom and compassion.^1^ Programmes based on relatively intensive cultivation of various forms of mindfulness and its application in daily life, referred to as Mindfulness-Based Interventions (MBIs), have produced impressive results in reducing symptoms across a wide range of mental and physical health problems, as well as among diverse populations around the world, and have become an important tool in health care.^1,2^

One of the most popular MBIs is Mindfulness-based stress reduction (MBSR). This standardised, eight-week group intervention was originally developed by Jon Kabat-Zinn at the University of Massachusetts in an effort to alleviate distress in chronic pain patients who had not achieved relief through traditional medical treatments.^3^ Since its inception, multiple research studies have demonstrated the effectiveness of MBSR in reducing a range of medical symptoms, including chronic pain, psoriasis, type-2 diabetes, cancer, fibromyalgia, arthritis, irritable bowel syndrome (IBS), obesity and the stress that accompanies daily life.^1,2,4^ The quality of much of this research is such that definitive inferences cannot be made, and as a result the next wave of research should be orientated towards more standardised research methodologies and randomised trials.^5^ In this regard, the data in physical disorders are relatively limited albeit promising. There is evidence of effectiveness of mindfulness interventions in relatively large randomised trials in women with IBS, distressed breast cancer survivors and fibromyalgia syndrome.^2^

Mindfulness-based approaches are effective in treating various psychological disorders.^6,7^ Randomised Controlled Trials (RCTs) indicate Mindfulness-Based Cognitive Therapy (MBCT) significantly reduces the risk of depressive relapse among individuals at risk^6,8,9^ and Mindfulness-Based Relapse Prevention (MBRP) improves outcomes for substance abuse.^2^ In addition, interventions may improve the quality of life across a range of stress-related conditions^2^ as well as for healthy individuals.^5^

Links between stress and health outcomes are based on findings that associate elevated stress levels with poor psychological well-being, physiological dysregulation and enhanced risk or worsening of organic disease.^10^

The MBSR intervention specifically targets stress reduction as a primary mechanism through which other health-related outcomes are impacted. Researchers^11^ hypothesise the potential mechanisms through which MBSR improves various health outcomes, which include:

- decreased perception of pain severity;
- increased ability to tolerate pain or disability;
- reduced stress, anxiety or depression;
- diminished usage of, and thereby reduced adverse effects of, analgesic, anxiolytic or antidepressant medication;
- enhanced ability to reflect on choices regarding medical treatments (e.g. decision to seek a second opinion);
- improved adherence to medical treatments;
- increased motivation for lifestyle changes involving diet, physical activity, smoking cessation or other behaviours;
- enriched interpersonal relationships and social connectedness; and
- alterations in biological pathways affecting health, such as the autonomic nervous system, neuroendocrine function and the immune system.

Most MBSR has been conducted in high-resourced settings, predominantly in North America and Europe. The importance of reducing the impact of stress and its health consequences in South Africa presents a significant challenge. Although MBIs have been implemented in various settings in South Africa, to date only one study (using MBCT) has measured the clinical and neurobiological impacts of MBCT on people with bipolar disorder.^12^ Therefore, to explore the potential impact of an eight-week MBSR on the health outcomes of a more clinically heterogeneous sample of South Africans, this study analysed clinical records of participants who completed an MBSR programme in Cape Town. Specifically, this study sought to investigate the impact of MBSR on the mindfulness of participants and whether this impact affected changes in medical symptoms, stress or mood.

## Methods

### Study design

A retrospective analysis was performed on data extracted from clinical records of 276 participants who completed an MBSR programme facilitated by two of the authors between 2004 and 2012. The records consisted of mindfulness, stress, mood and medical symptom questionnaires completed by participants before beginning the first session of the intervention and at the end of the final session.

### Setting

The MBSR programme was conducted in a private medical facility in Cape Town, South Africa, between 2004 and 2012. All participants in the MBSR programme self-selected, after referral either by their health professional or a previous participant of the programme and paid to participate in this therapeutic intervention for a variety of self-reported reasons.

### Study population and sampling strategy

The records of all participants who completed the MBSR course during the period of interest and completed both the pre-intervention and post-intervention measures of interest were eligible for inclusion in this study. While 387 participants completed the MBSR course, only 386 consented for their data to be used, and 110 were excluded because of incomplete post-intervention questionnaires. Thus, data from 276 participants were used for the analysis.

### Intervention

The MBSR programme is delivered by an MBSR facilitator, in group sessions of up to 25 participants. The intervention duration is 8 weeks, with weekly two and a half hour sessions and an 8 h (one day) retreat in week six. MBSR teaches four main mindfulness practices: (1) body scan, a process of moving attention through the body; (2) mindful movement, consisting of gentle yoga stretches to develop awareness of body in movement; (3) sitting meditation; and (4) walking meditation. Inquiry and didactic teaching is also a part of MBSR and allows for some detailed exploration of participants’ experiences. Daily homework of guided meditation practice using MP3s (45 min per day) of the practices is encouraged.

### Data collection

#### Mindfulness

Two measures of mindfulness were used. For records from 2004 to 2006, the Mindful Attention Awareness Scale (MAAS) was used and completed by 48 participants. For records from 2007, the Kentucky Inventory of Mindfulness Skills (KIMS) was used and 228 participants completed this measure. Both are valid measures of mindfulness and can be used interchangeably; thus, we included all participants who completed either the MAAS or the KIMS. The shift from MAAS to the KIMS reflects the general trend in the research literature which increasingly used the latter as a more granulated measure of the qualities of mindfulness.

The MAAS^13^ is a 15-item, single-factor instrument measured on a Likert scale (1 = almost always, 2 = very frequently, 3 = somewhat frequently, 4 = somewhat infrequently, 5 = very infrequently, and 6 = almost never). This measure uses a cognitive framework to conceptualise mindfulness and describes it as an open or receptive attention and awareness of present events and experience. The MAAS is also designed to measure innate mindfulness ability, as well as an individual’s ability to maintain the high levels of consciousness necessary for mindful practice. Items were summed to calculate a final score, where higher scores indicate greater mindfulness. The authors of the instrument report good to excellent reliability (Cronbach’s α = 0.96 and test–retest reliability of approximately 0. 81).

The KIMS^14^ is a 39-item, self-report measure comprising four mindfulness subscales (Observe, Describe, Acting with Awareness, and Accepting or Allowing without Judgement). Items are rated on a 5-point Likert-type scale (where 1 = never or very rarely true, 2 = rarely true, 3 = sometimes true, 4 = often true, and 5 = very often or always true) and aggregated to get subscale totals and the total KIMS score. Higher scores indicate greater mindfulness (minimum = 39; maximum = 195).

The authors of the KIMS have found the measure to have good internal consistency (Cronbach’s α = 0.83 to 0.91) and validity.

#### Stress

The Perceived Stress Scale (PSS)^15^ is a 10-item, self-report instrument measuring the degree to which situations in one’s life in the past month are perceived as unpredictable, uncontrollable and overwhelming. Items are rated on a 5-point Likert scale (0 = never, 1 = almost never, 2 = sometimes, 3 = fairly often, and 4 = very often). Items are summed to calculate a total score, where a higher score reflects an increased degree of stress (minimum = 0; maximum = 40). The instrument is widely used and considered to be a valid, reliable and comprehensive measure of stress^16^. The PSS has fair to good internal consistency (Cronbach’s α = 0.79) and test–retest reliability.

#### Mood

The Profile of Mood States (POMS)^17^ is a self-report measure with six subscales: tension-anxiety, depression-dejection, anger-hostility, vigour, fatigue and confusion-bewilderment. Participants respond to 65 adjectives reflecting their appropriate mood states in the preceding week. The adjectives include positive (e.g. carefree, cheerful and lively) and negative (e.g. gloomy, deceived and guilty) mood states. Each item is measured on a 5-point Likert-type scale (0 = not at all, 1 = a little, 2 = moderately, 3 = quite a bit, and 4 = extremely). The POMS has been used extensively with a wide range of populations and has been found to be valid and with excellent reliability (Cronbach’s α = 0.91)^18^. In this study, five subscales were summed to create a negative mood composite (minimum = 0; maximum = 184; where high scores indicate more negative moods), while scores on the vigour subscale were used to represent positive mood (minimum = 0; maximum = 32).

### Medical symptoms

The Medical Symptom Checklist (MSCL) was used to assess the number of medical symptoms pre and post-treatment. The 90-item inventory also examines psychological distress, anxiety and depression. Participants respond yes or no to each symptom. This instrument has frequently been used in mindfulness research, notably by Kabat-Zinn to assess medical symptom response in his pioneering MBSR research^3^. We divided the MSCL into two subscales for this study: the number of psychology-related symptoms and total number of medical symptoms.

#### Data analysis

A statistical analysis of the data were conducted using IBM SPSS Statistics 21. Dependent t-tests or Wilcoxon signed-rank tests were used to analyse pre and post-MBSR results. Mindfulness outcomes on the MAAS and KIMS were analysed separately. Pearson’s correlations were used to examine the association between changes in mindfulness and changes in mood, stress and medical symptoms. Where participants left out individual items on the questionnaires, the missing values were computed using linear interpolation (271 cases in 160 questions, amounting to 0.4% of the data). Where a participant left out the majority of a questionnaire (1 KIMS Post, 2 PSS Post and 1 POMS Post, from different participants), data imputation was not appropriate, and the items were left blank. The data for all the measures were normally distributed except for the POMS negative, which was positively skewed at pre and post-measurement times. The POMS negative changes were therefore analysed using a Wilcoxon signed-rank test. Dependent *t*-tests were used to analyse changes in other measures. To adjust for the number of comparisons done, a more conservative alpha of 0.01 was used throughout.

## Ethical consideration

Permission to conduct this study was granted by the Medical Research Council of South Africa’s Ethics Committee. Informed consent was sought from all eligible participants through email notification (all eligible participants had valid email addresses on record). The MBSR facilitators were both health professionals. Anonymity, confidentiality and withdrawal from the study without penalty were assured. Only data from the four measures of interest (detailed above) and attendance data were entered into a database; no identifying information or other private health information was extracted from the clinical records. Permit number MRC IRB with EC001-2/2013.

## Results

Participants’ pre- and post-MBSR scores for mindfulness and health outcomes are shown in Table 1. As a measure of mindfulness, 228 participants completed the KIMS and 48 completed the MAAS. Before intervention, participants, on average, had a KIMS score of 118.45 (standard deviation [SD] = 18.83; out of a possible 156) or a MAAS score of 47.15 (SD = 12.41; out of 90), where higher scores indicated greater mindfulness. Average POMS positive and negative mood scores were 15.54 (SD = 6.21) and 65.66 (SD = 31.00), respectively (negative scores were higher because of more subscales). Perceived stress scores were high before intervention: participants scored, on average, 20.41 out of 40 on the PSS (SD = 6.52). Reported medical symptoms ranged from 0 to 60, and reported psychological symptoms ranged between 0 and 18. The average number of symptoms reported was 19 (SD = 11), and on average participants reported experiencing 7 (SD = 4) psychological symptoms before intervention.

**TABLE 1:** Descriptive statistics for the mindfulness, mood, stress and medical symptom scales.

Statistic	KIMS		MAAS		POMS				PSS		MSCL			
					Positive		Negative				Total		Psychological	
	Pre	Post	Pre	Post	Pre	Post	Pre	Post	Pre	Post	Pre	Post	Pre	Post
*n*	228	228	48	48	275	276	275	276	229	227	275	275	275	275
Minimum	70	78	23	38	1	2	8	4	3	5	0	0	0	0
Maximum	167	172	74	82	31	32	161	169	36	37	62	60	18	17
Mean	118.45	133.59	47.15	60.13	15.54	18.32	65.66	46.79	20.41	15.92	18.76	13.51	7.26	4.71
SD	18.83	16.48	12.41	10.13	6.21	5.72	31.00	30.22	6.52	6.41	11.12	11.24	4.36	4.30

KIMS, Kentucky Inventory of Mindfulness Skills; MAAS, Mindful Attention Awareness Scale; MSCL, Medical Symptoms Checklist; PSS, Perceived Stress Scale; POMS, Profile of Mood States; SD, standard deviation.

### Changes in mindfulness, mood, stress and medical symptoms

As shown in Table 1, mindfulness scores were on average significantly higher post-MBSR on both the KIMS (*t* [227] = −14.63, *p* < 0.001) and the MAAS (*t* [46] = −7.31, *p* < 0.001). Negative mood was significantly lower post-MBSR (*Z* = −8.56, *p* < 0.001), while positive mood was significantly higher post-MBSR (*t* [274] = −7.08, *p* < 0.001). Stress scores were also significantly lower post-MBSR (*t* [226] = 10.16, *p* < 0.001). The total reported medical symptoms were significantly lower post-MBSR (*t* [273] = 10.36, *p* < 0.001), as were psychological symptoms (*t* [273] = 9.91, *p* < 0.001).

### Correlation between changes in mindfulness and changes in mood, stress and medical symptoms

Increased mindfulness scores from the KIMS were statistically significantly related to decreased negative mood, increased positive mood, decreased stress and decreased medical and psychological symptoms (see Table 2). A similar pattern of correlations was seen when using the MAAS, although the only statistically significant correlation was between change in MAAS scores and change in medical symptoms. Data for the correlation between the PSS and MAAS are not available, as the PSS was not administered during the time that the MAAS was administered.

**TABLE 2:** Correlation between changes in mindfulness and changes in mood, stress and medical symptoms.

Change scores	POMS positive change score	POMS negative change Score	PSS change score	MSCL total change score	MSCL psych change score
KIMS†	0.213**	−0.370**	−0.463**	−0.216**	−0.351**
MAAS‡	0.132	−0.314	N/A	−0.380*	−0.291

KIMS, Kentucky Inventory of Mindfulness; MAAS, Mindful Attention Awareness Scale; MSCL, Medical Symptoms Checklist; MSCL Psych, psychological symptoms; PSS, Perceived Stress Scale; POMS, Profile of Mood States; N/A, not available.

† , *N* = 226; ‡, *N* = 46.

* , *p* < 0.01;

** , *p* ≤ 0.001.

## Discussion

As the first study of MBSR’s impact on a clinical sample in South Africa, this study provides significant preliminary statistical evidence for the intervention’s value in an urban South African setting, with respect to stress reduction and improvement in medical symptoms and mood. Overall, participants showed statistically significant improvement in mindfulness after completing the MBSR and these changes were associated with gains in positive mood, decreases in negative mood, stress reduction, and medical and psychological symptom reduction. These improvements are consistent with findings of international studies.^19^

The trends observed in this study, and international studies, may be explained by other research findings that seek to understand the mechanisms of change. Firstly, EEG results indicate greater left anterior activation after an MBSR intervention,^20^ areas of the brain that are thought to be associated with greater disposition to positive affect. Secondly, MBSR presents participants with opportunities to practice different internal responses to difficult and distressing experiences, which allows individuals to better self-regulate and increase distress tolerance over time.^21^

This study has several limitations. The sample was self-selected from a moderate to high-income population and thus generalising the findings to the broader South African population is restricted. Furthermore, the participants may have been particularly open to MBSR and the mindfulness practices it promotes because they were motivated to seek this particular therapeutic option. As a retrospective record review, this study design did not include a control group or measures of potential confounding factors (e.g. socio-demographic background information). Of the sample, 110 participants did not complete the post-intervention questionnaires. Questionnaires were sometimes taken home by participants at the end of programmes, with the promise of completion, as opposed to being completed on site. It is possible that incomplete post-intervention surveys could have introduced bias. Furthermore, there was no measurement of homework compliance, both of which may have impacted results. However, our study did not seek to assess how compliance, or the number of hours practised influenced stress and reported symptoms, but rather whether simple course attendance influenced psychological and medical outcomes. Despite some limitations, this study provides important preliminary evidence based on a South African subgroup among whom the effectiveness of MBSR had not yet been tested.

## Conclusion

The results of this study provide preliminary support for the use of MBSR as a valuable intervention to reduce stress, improve mood states and decrease medical symptoms in the South African context, substantiating the need for ongoing, more context-specific MBSR research. Future studies should explore the acceptability and feasibility of MBSR among a representative and randomly selected sample of the broader South African population with a wait list control in order to inform potential adaptations that can be evaluated for efficacy and effectiveness.
